# Dr. J. N. Farrar’s Work on Irregularities

**Published:** 1890-11

**Authors:** 


### DR. J. N. FARRAR'S WORK ON IRREGULARITIES.

We take special pleasure in being able to announce that the first volume of this remarkable work will be ready for distribution in a few days. A full review of it will be given in our next number.

That this year should witness the publishing of two such books as Farrer's "Irregularities of the Teeth" and Miller's "Micro-Organisms of the Oral Cavity," the first after seven years of labor, and the latter ten, is an epoch in dentistry and calls for special notice. They furnish the best illustration of the change in the general trend of professional thought and work towards a higher standard.

